# Improved diagnosis of colorectal cancer using combined biomarkers including *Fusobacterium nucleatum*, fecal occult blood, transferrin, CEA, CA19‐9, gender, and age

**DOI:** 10.1002/cam4.6067

**Published:** 2023-05-10

**Authors:** Ran Zhao, Dongge Xia, Yingwei Chen, Zhentian Kai, Fangying Ruan, Chaoran Xia, Jingkai Gong, Jun Wu, Xueliang Wang

**Affiliations:** ^1^ Shanghai Center for Clinical Laboratory Shanghai China; ^2^ Department of Clinical Laboratory Shanghai General Hospital, Shanghai Jiao Tong University School of Medicine Shanghai China; ^3^ Zhejiang Shaoxing Topgen Biomedical Technology CO., LTD. Shanghai China; ^4^ Department of Clinical Laboratory Shanghai General Hospital Jiading Branch, Shanghai Jiao Tong University School of Medicine Shanghai China

**Keywords:** biomarker, colorectal cancer, *Fusobacterium nucleatum*, machine learning, risk model, screening

## Abstract

**Background:**

Conventional blood and stool tests are normally used for early screening of colorectal cancer (CRC) but the accuracy and efficiency remain to be improved. Recent findings suggest *Fusobacterium nucleatum* to be a biomarker for CRC. This study evaluated the role of *F. nucleatum* and developed CRC diagnostic models by combining *F. nucleatum* with fecal occult blood (FOB), transferrin (TRF), carcinoembryonic antigen (CEA), carbohydrate antigen 19‐9 (CA19‐9), gender, and age.

**Materials and Methods:**

Candidates including 71 healthy individuals and 59 CRC patients were recruited. Abundance of *F. nucleatum* in stool or tissue samples was measured by quantitative real‐time PCR. CEA, CA19‐9, TRF, and FOB were measured in parallel. These biomarkers together with genders and ages were the seven parameters used to develop CRC diagnostic models. Ten different machine learning algorithms were tested to achieve the best performance.

**Results:**

Fecal *F. nucleatum* abundance was found significantly higher in CRC group compared to healthy group (*p* = 0.0005). Among the CRC patients, *F. nucleatum* abundance in tumor tissue was significantly higher than that in paracancerous tissue (*p* = 0.0087). CRC diagnostic models using different parameters were generated based on Logistic Regression algorithm, which showed good performance. The area under the curve (AUC) score of fecal *F. nucleatum* as the single diagnostic biomarker was 0.68 while the accuracy was 0.56. The diagnostic performance was obviously improved with the highest AUC (0.93) and accuracy (0.87) achieved when using all the 7 clinical parameters. The combination *F. nucleatum* + FOB + gender + age had the second highest AUC (0.92) and accuracy (0.85). A more utilitarian model using *F. nucleatum* + FOB showed relatively high AUC at 0.86 and accuracy at 0.81.

**Conclusions:**

*F. nucleatum* is valuable for CRC diagnosis. Combination of different clinical parameters could significantly improve CRC diagnostic performance. The combination *F. nucleatum* + FOB + gender + age may be an effective and noninvasive method for clinical application.

## Graphical abstract

*Fusobacterium nucleatum* is valuable for CRC diagnosis. Combination of different clinical parameters could achieve high CRC diagnostic performance with the AUC at 0.93 and accuracy at 0.87 based on the generated Logistic Regression model. A more utilitarian model using the two biomarkers fecal *F. nucleatum* and FOB may be an effective and noninvasive method for clinical application.

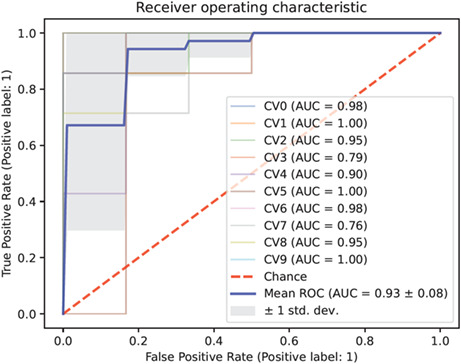

## 1 INTRODUCTION

Colorectal cancer (CRC) is the world's fourth most deadly cancer and has the highest rates of incidence in developed countries.^1^, ^2^ The 5‐year survival rate of CRC patients is above 90% for Stage I and declines to 11%–15% for Stage IV.^3^, ^4^ Early diagnosis of CRC is crucial for timely tumor treatment and is the key to reducing the incidence and mortality associated with the disease.^5^

Currently, early screening and monitoring of CRC rely on conventional tumor markers including fecal occult blood (FOB), fecal transferrin (TRF), carcinoembryonic antigen (CEA), and carbohydrate antigen 19‐9 (CA19‐9).^6^, ^7^, ^8^ Fecal occult blood test (FOBT) is the most widely used noninvasive approach, while it has drawbacks such as poor specificity and low positive predictive value.^8^, ^9^, ^10^ Previous clinical studies found that TRF was as useful as FOB in diagnosing colorectal diseases.^11^, ^12^ However, TRF and FOBT results cannot identify CRC lesions that are not accompanied by bleeding.^13^ Moreover, both CRC and polyps can release trace amounts of blood in stool, and this may lead to false positive CRC screening results.^14^ Serum CEA and CA19‐9 are well‐known tumor markers for CRC treatment monitoring, especially in chemotherapy patients.^6^ Repeated elevations of CEA or CA19‐9 levels after surgery may suggest the presence of residual disease or recurrent risks.^15^ However, CEA and CA 19‐9 have not been recommended as screening markers for CRC, which may be due to their low sensitivities and specificity, as other diseases could also lead to elevated levels of these clinical parameters.

Human gut microbiota plays an important role in host physiology, immunity, metabolism, and nutrition.^16^ Alterations of the gastrointestinal microbial community are related to different health and disease status, such as cancer, obesity, and a variety of bowel disorders.^17^ Increasing evidence links certain microorganisms in the gut microbiota with CRC together. Metagenomic analyses suggest that the intestinal bacteria *Fusobacterium nucleatum* is implicated in the development and progression of CRC.^18^, ^19^, ^20^, ^21^ Previous studies indicate that symbiotic *Fusobacterium* spp. were enriched in CRC tissues compared to healthy tissues.^22^ *F. nucleatum* may potentiate intestinal tumorigenesis by modulating the tumor‐immune microenvironment, activating the autophagy signaling pathway, or through other mechanisms.^21^, ^23^ These findings give new opportunities to take advantage of microbial biomarkers for clinical applications, such as the detection of fecal *F. nucleatum* may provide much‐needed progress in the ability to screen CRC noninvasively.^20^, ^24^ However, current detection method of *F. nucleatum* varies from one another. Developing diagnostics and therapeutics for *F. nucleatum* remain challenged.^20^ The value of *F. nucleatum* as a screening, prognostic or predictive biomarker for CRC has not been fully defined in clinical applications.

Although biomarkers have many attractive features as cancer screening tests, the major concern for CRC screening or early diagnosis using different clinical biomarkers is their insufficient sensitivity and/or specificity.^7^ Due to the low prevalence of cancer in the general population, most biomarkers being used alone have a low positive predictive value in screening asymptomatic populations.^10^ Therefore, developing a diagnostic model using a panel of tumor markers may be a feasible approach that can potentially increase the efficiency of CRC screening.^7^ However, current approaches using fecal‐/blood‐based biomarker panels are not cost‐effective and the detection accuracies remain unsatisfactory.^25^, ^26^ CRC‐related gut microbe markers are under development in order to find out the best combination of biomarkers that is cost‐effective and maximize the screening sensitivity and specificity.^27^ Herein, we present the potential role of *F. nucleatum* in CRC screening and develop a combined biomarker model using *F. nucleatum* with other fecal‐/blood‐based markers including FOB, TRF, CEA, and CA19‐9, as well as personal characteristics including gender and age. The generated utilitarian model may provide a new strategy for early CRC diagnosis.

## 2 MATERIALS AND METHODS

### 2.1 Patients and sampling

A total of 130 individuals from Shanghai General Hospital during January 2021 to July 2022 were retrospectively analyzed, including 59 CRC patients and 71 healthy controls. The CRC patients were diagnosed and determined according to the International Union Against Cancer (UICC)/American Cancer Society (AJCC). Stages of the CRC patients included 1 Stage 0 (TisN0M0), 3 Stage II, 21 Stage III, 20 Stage IV, and 14 unknown. Control subjects were selected randomly from individuals undergoing health screening. Participants who used antibiotics or probiotics within 1 month before sampling were excluded. Stool and serum samples were collected in both healthy and CRC groups before surgeries. Tissue samples were collected in CRC patients during surgeries. Serum samples were processed for CEA and CA19‐9 measurement immediately using the Beckman Coulter DxI 800 immunoassay system. A portion of fresh fecal samples was used for FOB and fecal TRF measurement by chemical kit (BASO) according to the manufacturer's instructions. The rest fecal samples and the tissue samples were stored into RNAlater solution (Thermo Fisher Scientific) at −80°C before DNA extraction. Patient demographic data including age and gender were recorded.

### 2.2 Ethical approval

This study was approved and supervised by the ethical committees of Shanghai Center for Clinical Laboratory and Shanghai General Hospital (File Nos. 202003 and 221105). All experiments were conducted in accordance with the relevant guidelines and regulations. Written informed consent was obtained from all participants in the study.

### 2.3 Measurement of *F. nucleatum* abundance

#### 2.3.1 DNA extraction

Fecal genomic DNA was isolated using the QIAamp DNA Stool Mini Kit (Qiagen). Tissue DNA was extracted from tumor tissue and paracancerous tissue samples using the DNeasy Blood & Tissue kit (Qiagen). The extracted DNA was quantified by a NanoDrop One Microvolume UV‐Vis Spectrophotometer (Thermo Fisher Scientific). All DNA samples were stored at −20°C before use.

#### 2.3.2 Primers and probes

A quantitative real‐time PCR (qPCR) system was designed for detection of the *nusG* gene of *F. nucleatum* and the 16S rRNA conserved regions. The oligonucleotide primers and probes were synthesized by Sango Biotech Co., Ltd. The *F. nucleatum* forward primer sequence was 5′‐TCAAGAGGGACTCGAACC‐3′, and reverse primer sequence was 5′‐CCTGCATGTGTTGTTAACTG‐3′. The amplicon size was 86 bp. The sequence of *F. nucleatum* probe was FAM‐5′‐GGAGACCGATGCTCTACCAATTGAG‐3′‐ BHQ1. The 16S rRNA forward 5′‐GMAACRCGARGAACCTTACC‐3′ and 16S rRNA reverse 5′‐GCGCTCGTTRCGGGACTTAA‐3′ primers were used for amplification of total bacteria and also as the internal control, with the 16S rRNA probe ROX‐5′‐ GCATGGYTGTCGTCAGCTCGTGT‐3′‐ BHQ2.

#### 2.3.3 Optimization of the qPCR reactions

*F. nucleatum* abundance in all extracted DNA samples was analyzed by qPCR. Reactions were performed in a total volume of 25 μL containing 12.5 μL 2 × THUNDERBIRD® Probe qPCR mix (Toyobo (Shanghai) Biotech Co., Ltd.), 0.2–0.8 μM primers, 0.1–0.4 μM probes, and 5 μL DNA templates.

Experiments were conducted using a QuantStudio™ 5 Real‐Time PCR System (Applied Biosystems™, Thermo Fisher). All reactions were detected under the following conditions: Pre‐denaturation with one cycle of 95°C for 10 min; denaturation at 95°C 15 s followed by extension/annealing at 60°C 1 min, repeating 40 cycles; fluorescence signal data were collected at the extension step.

Relative abundance of *F. nucleatum* was calculated by the 2^−ΔCt^ method as compared to the total bacteria (16S rRNA), where △Ct is the difference between Ct values of *F. nucleatum* and 16S rRNA.

### 2.4 Development of CRC diagnostic models

Seven clinical parameters from all participants in this study were included for modeling, namely CRC‐related biomarkers including fecal *F. nucleatum*, CA19‐9, CEA, FOB, and TRF, as well as personal characteristics such as gender and age of the participants. Ten different machine learning algorithms were tested to identify the best diagnostic model with good performance. To ensure the reliability of the developed model and improve the model stability, a 10‐fold cross‐validation method was used to further optimize the model parameters.

### 2.5 Statistical analysis

Statistical analysis was carried out using R 3.5.1. A *p* value less than 0.05 was considered statistically significant. Mann–Whitney *U*‐test or Wilcoxon signed rank test was used to analyze the differences in *F. nucleatum* abundance, CEA and CA19‐9 levels. Independent *t*‐test or chi‐squared test was used to compare differences in other characteristics between groups.

## 3 RESULTS

### 3.1 General characteristics

Table 1 gives a general description of the 59 CRC patients (CRC) and 71 healthy candidates (Healthy) enrolled in this study. Means with standard deviations or case numbers with percentages were calculated for each group and shown in the table. The two groups had an equal distribution of genders, while the CRC patient group had higher average age, CEA and CA19‐9 levels, as well as more FOB and TRF positive results compared to the control group (*p* < 0.05).

**TABLE 1:** General characteristics of the CRC patients and healthy controls.

	Healthy	CRC	Test	*p* value
Gender				
Male	45 (63.38%)	34 (57.63%)	Chi‐square (Pearson = 0.447)	0.504
Female	26 (36.62%)	25 (42.37%)		
Age	46.34 ± 12.23	63.03 ± 11.77	*t*‐test	<0.001
CEA	1.59 ± 0.99	44.75 ± 128.64	*t*‐test	0.002
CA19‐9	5.86 ± 4.18	92.92 ± 190.23	*t*‐test	0.014
FOB				
Positive	0 (0%)	17 (28.8%)	Chi‐square (Pearson = 57.636)	<0.001
Negative	71 (100%)	24 (40.7%)		
Unknown	0 (0%)	18 (30.5%)		
TRF				
Positive	0 (0%)	13 (22.0%)	Chi‐square (Pearson = 48.986)	<0.001
Negative	71 (100%)	28 (47.5%)		
Unknown	0 (0%)	18 (30.5%)		

### 3.2 *F. nucleatum* was enriched in tumor tissue in CRC patients

**FIGURE 1:**
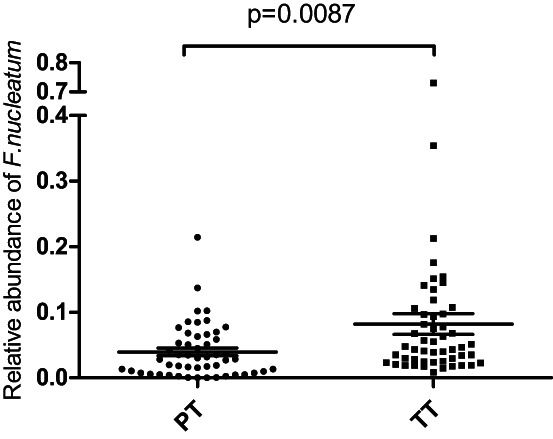
Relative abundance of *Fusobacterium nucleatum* in the tumor tissues (TT) was significantly higher than that of the paired paracancerous tissues (PT) in CRC patients (Wilcoxon signed rank test, *n* = 51, *p* = 0.0087). Relative abundance of *F. nucleatum* was calculated by the 2^−ΔCt^ method as compared to the total bacteria (16S rRNA). The middle line with error bars on the graph indicates the mean and standard error of each group.

The abundance of *F. nucleatum* in the tumor tissues and paracancerous tissues was investigated in 51 CRC patients (Figure 1), as 8 CRC patients failed to collect tissue samples. The average *F. nucleatum* abundance with standard error (SE) of the tumor tissues and the paracancerous tissues were 0.082 ± 0.016 and 0.040 ± 0.006, respectively. *F. nucleatum* abundance in the tumor tissue was significantly higher than that in the paracancerous tissue (Wilcoxon signed rank test, *p* = 0.0087).

### 3.3 Fecal *F. nucleatum* abundance was higher in CRC patients but not related to age

**FIGURE 2:**
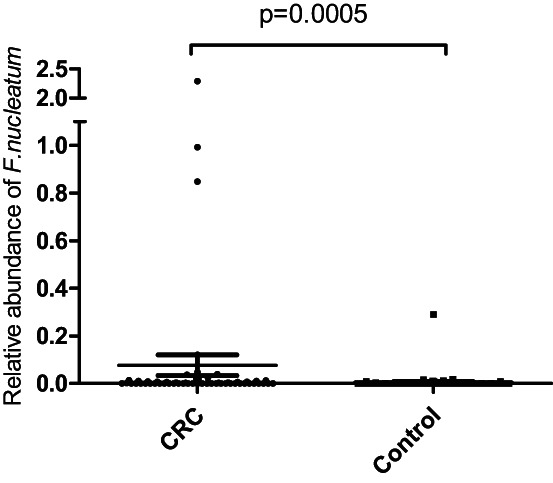
Relative abundance of *Fusobacterium nucleatum* in fecal samples of CRC patients (CRC) was significantly higher than that of the control group (Control) (Mann–Whitney *U*‐test, *p* = 0.0005). Relative abundance of *F. nucleatum* was calculated by the 2^−ΔCt^ method as compared to the total bacteria (16S rRNA). The middle line with error bars on the graph indicates the mean and standard error of each group.

Fecal *F. nucleatum* abundance was measured and compared between the CRC group and the control group, as shown in Figure 2. The average *F. nucleatum* abundance with SE in the CRC group (*n* = 59) was 0.077 ± 0.044 and the control group (*n* = 71) was 0.0055 ± 0.004. A significantly higher level of *F. nucleatum* was found in CRC patients than in the controls (Mann–Whitney *U*‐test, *p* = 0.0005).

As the CRC group has higher average age than the control group (Table 1), further analysis was done to compare the fecal *F. nucleatum* abundance between different age groups. Candidates with ages above the mean age in each group were set as high‐age‐group while those with ages below the mean of each group were set as low‐age‐group. The abundance of *F. nucleatum* in the high‐age‐group has no difference from that in the low‐age‐group (Mann–Whitney *U*‐test, *p* = 0.428).

### 3.4 Diagnostic model based on Logistic Regression algorithm shows good performance

The seven clinical parameters including FOB, TRF, CEA, CA19‐9, fecal *F. nucleatum* amount as well as gender and age were used to generate an applicable diagnostic strategy for CRC screening. Different machine learning algorithms were employed to develop diagnostic models, and the performance was compared as shown in Table 2. Among them, the Logistic Regression model has the highest accuracy (0.86) and a relatively high area under the curve (AUC) score (0.93). The Ridge Classifier, Random Forest Classifier, and Extra Trees Classifier models showed similar or slightly higher AUC scores compared to the Logistic Regression model. However, the Logistic Regression model is more suitable for research with a limited sample size due to its better interpretability which could considerably avoid overfitting problems. Therefore, the Logistic Regression model was eventually selected in this study.

**TABLE 2:** Performance of different machine learning models.

Model	Accuracy	AUC	Recall	Precision	F1 score	Kappa	Matthews correlation coefficient
Logistic Regression	0.86	0.93	0.92	0.87	0.88	0.72	0.74
Ridge Classifier	0.86	1.00	0.95	0.84	0.89	0.70	0.72
Linear Discriminant Analysis	0.86	0.91	0.96	0.84	0.89	0.69	0.72
Random Forest Classifier	0.84	0.93	0.91	0.86	0.87	0.68	0.70
Gradient Boosting Classifier	0.84	0.92	0.91	0.86	0.87	0.68	0.70
Extra Trees Classifier	0.84	0.94	0.89	0.87	0.87	0.68	0.70
Decision Tree Classifier	0.82	0.81	0.88	0.83	0.85	0.63	0.65
Naive Bayes	0.81	0.91	0.95	0.79	0.86	0.60	0.64
K Neighbors Classifier	0.80	0.87	0.86	0.82	0.83	0.58	0.60
Ada Boost Classifier	0.80	0.88	0.85	0.83	0.83	0.59	0.60

**FIGURE 3:**
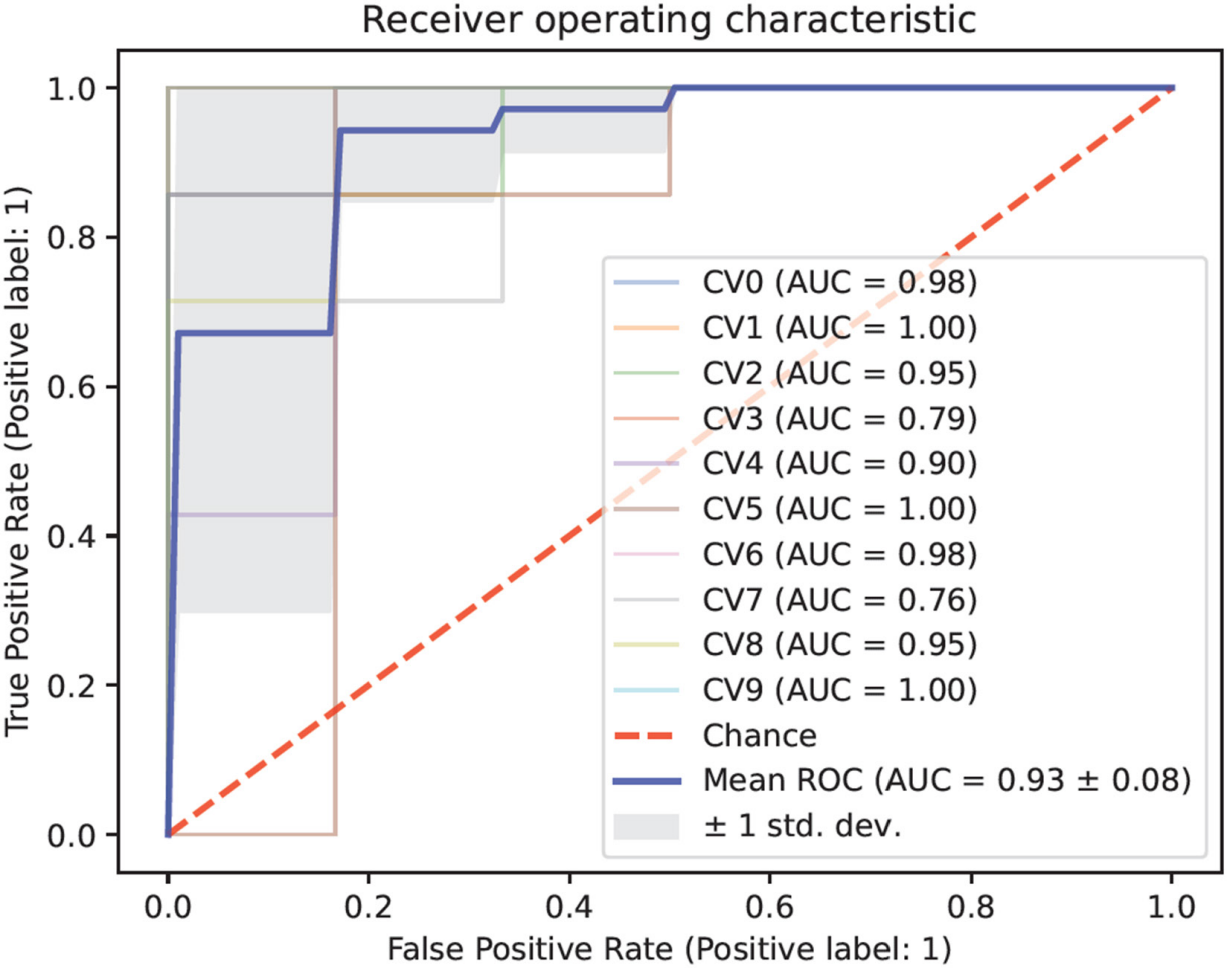
Receiver operating characteristic (ROC) curve of the Logistic Regression model shows good performance for CRC screening. The average AUC could achieve as high as 0.93. The 10‐fold cross‐validation method was used to optimize the model: 10 different curves (CV0–CV9 in different colors) were calculated, and the blue one (mean ROC) was the average. Gray area shows the standard deviation (±1 std. dev.).

Figure 3 gives the receiver operating characteristic (ROC) curve of the generated Logistic Regression model for CRC diagnosis using the 7 clinical parameters including fecal *F. nucleatum*, FOB, TRF, CEA, CA19‐9, gender, and age of the whole data set. Model parameters were optimized using the 10‐fold cross‐validation method to improve the model stabilities. The mean AUC score with standard deviation was 0.93 ± 0.08.

### 3.5 Diagnostic performance of different biomarker combinations

**FIGURE 4:**
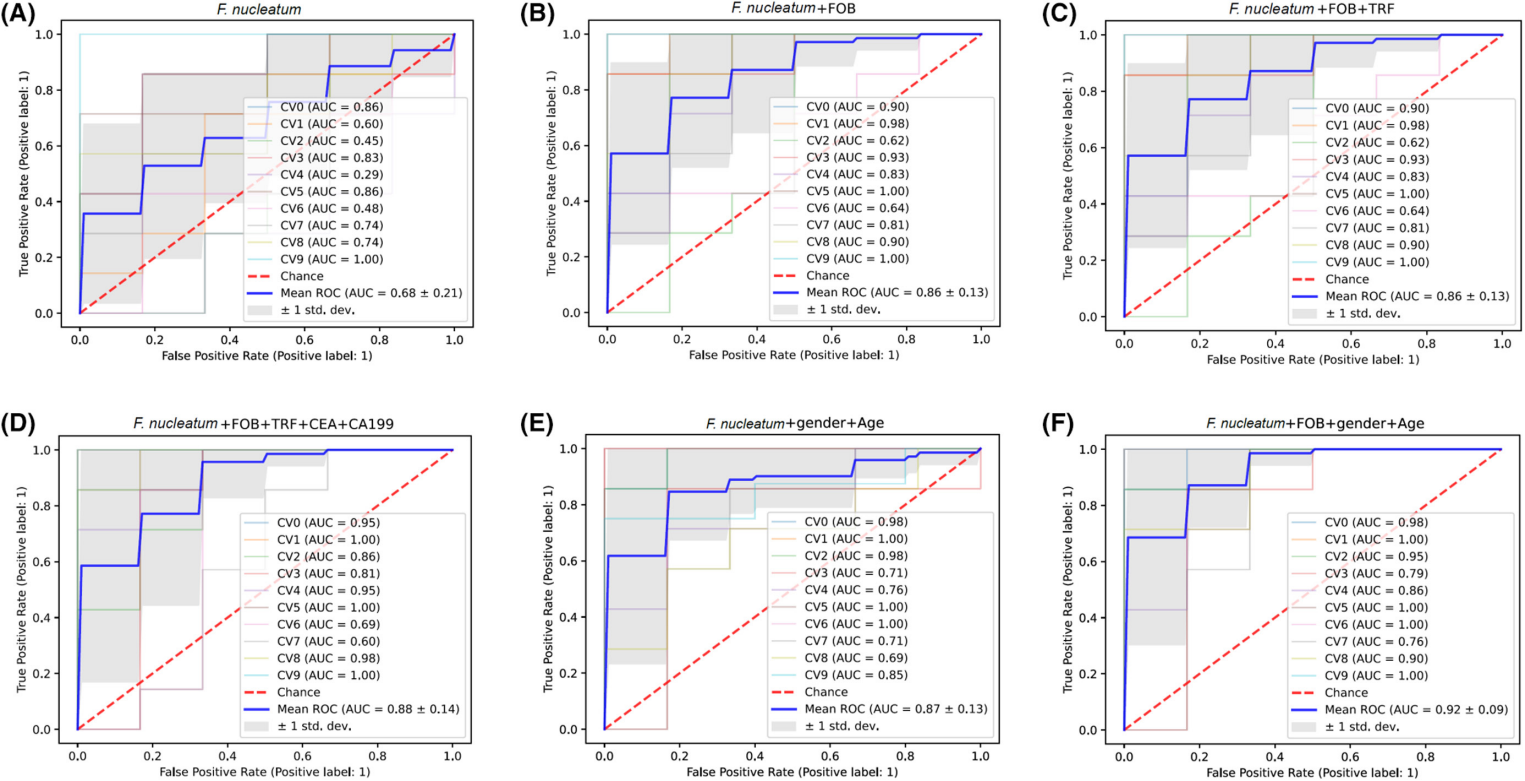
ROC curves of the diagnostic models using different biomarker combinations. (A) The AUC of *Fusobacterium nucleatum* as the single biomarker model was 0.68; (B) the AUC of the combination *F. nucleatum* + FOB was 0.86; (C) the AUC of the combination *F. nucleatum* + FOB + TRF was 0.86; (D) the AUC of the combination *F. nucleatum* + FOB + TRF + CEA + CA19‐9 was 0.88; (E) the AUC of the combination *F. nucleatum* + gender + age was 0.87; (F) the AUC of the combination *F. nucleatum* + FOB + gender + age was 0.92. The 10‐fold cross‐validation method was used to optimize the models; 10 different curves (CV0–CV9 in different colors) were calculated, and the blue one (mean ROC) was the average. Gray area shows the standard deviation (±1 std. dev.).

ROC analyses were performed to evaluate the diagnostic value of *F. nucleatum* or combinations of *F. nucleatum* with other CRC‐related markers such as FOB, TRF, CEA, and CA19‐9, as well as gender and age of the candidates. Performance of these combinations was illustrated in Table 3 and Figure 4. The AUC and accuracy scores of *F. nucleatum*‐single‐marker (Figure 4A) were 0.68 and 0.56, respectively, while when combined with FOB, the AUC and accuracy could be visibly improved up to 0.86 and 0.81 (Figure 4B). With more biomarkers used in the diagnostic model, the AUC and accuracy could be further improved. The 5‐biomarker combination *F.nucleatum* + FOB + TRF + CEA + CA19‐9 had an AUC at 0.88 and an accuracy score at 0.84 (Figure 4D).

**TABLE 3:** Diagnostic performance of different biomarker combinations.

	AUC^a^	Sensitivity^a^	Specificity^a^	Accuracy	Recall	Precision	F1 score	Kappa	Matthews correlation coefficient
*F. nucleatum*	0.68 ± 0.21	0.05 ± 0.11	0.99 ± 0.04	0.56	0.99	0.56	0.71	0.03	0.05
*F. nucleatum* + FOB	0.86 ± 0.13	0.60 ± 0.29	1.00 ± 0.00	0.81	1.00	0.77	0.86	0.61	0.66
*F. nucleatum* + FOB + TRF	0.86 ± 0.13	0.60 ± 0.29	1.00 ± 0.00	0.81	1.00	0.77	0.86	0.61	0.66
*F.nucleatum* + FOB + TRF + CEA + CA19‐9	0.88 ± 0.14	0.70 ± 0.22	0.96 ± 0.06	0.84	0.96	0.81	0.87	0.67	0.70
*F.nucleatum* + FOB + TRF + CEA + CA19‐9 + gender + age	0.93 ± 0.08	0.80 ± 0.21	0.92 ± 0.09	0.87	0.95	0.86	0.89	0.72	0.74
*F. nucleatum* + gender + age	0.87 ± 0.13	0.73 ± 0.27	0.74 ± 0.23	0.74	0.74	0.78	0.74	0.47	0.48
*F. nucleatum* + FOB + gender + age	0.92 ± 0.09	0.80 ± 0.18	0.90 ± 0.11	0.85	0.90	0.86	0.87	0.70	0.72

^a^ The model parameters were optimized using the 10‐fold cross‐validation method and calculated using the mean ± 1 standard deviation of the 10 different curves.

Furthermore, adding in personal characteristics such as gender and age could also increase the diagnostic performance in this model. The AUC and accuracy scores of *F. nucleatum* + gender + age (Figure 4E) were 0.87 and 0.74, respectively, while the combination *F. nucleatum* + FOB + gender+age (Figure 4F) had a very high AUC at 0.92 and accuracy at 0.85, which was just slightly lower than the highest AUC (0.93) and accuracy (0.87) scores, achieved when using all the 7 clinical parameters (Figure 3).

## 4 DISCUSSION

Screening of CRC has been shown to reduce incidence and mortality^3^ but CRC diagnosis usually relies on an invasive procedure such as a colonoscopy examination. This invasive application may have a modest risk of trauma to patients, and it is difficult to apply to a large‐scale population in routine physical examinations.^28^, ^29^ Hence, noninvasive CRC screening methods with low costs and better outcoming benefits are of great importance.^27^ Fecal indicators such as FOB and TRF as well as serological indicators such as CEA and CA19‐9 are commonly used clinical biomarkers for CRC.^6^, ^7^, ^13^ However, they have drawbacks such as low specificity and/or sensitivity.^9^, ^30^ Even more, the diagnostic effectiveness of different tumor markers varied widely.^10^, ^26^, ^31^, ^32^ Therefore, looking for other highly sensitive and effective biomarkers or biomarker combinations for noninvasive CRC screening is urgently needed for clinical practice.

Numerous studies have provided compelling evidence for the potential role of *F. nucleatum* in colorectal carcinogenesis and the outcomes of CRC patients.^20^ Detection of *F. nucleatum* might serve as a novel diagnostic tool for CRC.^24^ In this study, a fluorescence real‐time PCR method was developed and applied for *F. nucleatum* detection in both fecal and tumor tissue samples. Relative abundance of *F. nucleatum* in CRC patients and healthy controls was quantified using the optimized qPCR system. The result showed significantly higher abundance of *F. nucleatum* in tumor tissues than in paracancerous tissues. Moreover, fecal *F. nucleatum* levels in CRC patients were found significantly higher than that in healthy individuals. These results are consistent with previous reports,^33^, ^34^ which demonstrate again that *F. nucleatum* may be a useful biomarker for CRC detection. Measuring and targeting *F. nucleatum* will yield valuable insight into clinical screening and management of CRC patients. Considering that tissue‐based microbial markers are invasive and less accessible than stool‐based microbial markers, detection of fecal *F. nucleatum* can be applied as a noninvasive method to aid CRC diagnosis.

On the contrary, screening decisions may not be made based on only one indicator.^10^, ^35^ In the era of personalized medicine, sophisticated prediction software based on optimized algorithmic models with a panel of biomarkers may better guide screening results.^35^ In this paper, 10 different algorithmic models were developed and compared using the 5 fecal‐/blood‐based biomarkers including *F. nucleatum*, FOB, FOB, TRF, CEA, and CA19‐9, as well as two personal characteristics including gender and age. Among them, the Logistic Regression model using all these seven clinical parameters demonstrated good performance for CRC screening based on our current dataset. The diagnostic performance of *F. nucleatum* or combinations of *F. nucleatum* with other clinical indicators (FOB, TRF, CEA, CA19‐9, gender, and age) was subsequently evaluated. It seems that fecal *F. nucleatum* alone was not effective enough for CRC diagnosis as the AUC and accuracy scores were only 0.68 and 0.56, respectively. However, the AUC and accuracy could be visibly increased to 0.87 and 0.74 when combining the fecal *F. nucleatum* single biomarker with gender and age. This might be because personal characteristics such as gender and age are highly related to CRC risks. Incidence is higher in men than women and strongly increases with age.^3^ Adding in these parameters could increase the diagnostic performance of the developed model.

Moreover, combinations of two or more types of biomarkers are often used in clinical practices to improve the accuracy and efficiency of disease diagnosis.^10^ Our results demonstrate that better diagnostic performance could be achieved using combined clinical indicators compared to a single indicator. Based on the optimized Logistic Regression model, the 7‐parameter combination *F. nucleatum* + FOB + TRF + CEA + CA19‐9 + gender + age showed the highest AUC (0.93) and accuracy (0.87). The combination *F. nucleatum* + FOB + gender + age had the second highest AUC (0.92) and accuracy (0.85). Besides, *F. nucleatum* + FOB also showed high AUC and accuracy scores (0.86 and 0.81, respectively). Considering that only fecal specimens are required for detection of *F. nucleatum* and FOB, combination of these two biomarkers is much easier for clinical application. Therefore, application of fecal *F. nucleatum* detection combined with other clinical markers could increase the detection rate of CRC in normal individuals, and the combination *F. nucleatum* + FOB + gender + age may be the best choice in practical work.

Our results provide information on how to apply *F. nucleatum* as a new biomarker for the detection of CRC occurrence in clinical practice. However, different pathological features, such as stages, differentiations, and lymph node metastasis of the CRC patients, were not distinguished in this study. Besides, only patients within the past 2 years were taken from one hospital. As the risk factors for CRC may differ a lot among different populations or habitations, the diagnostic performance could be different when using the generated diagnostic models in other areas. Therefore, a large population study should be finished for further validation of the diagnostic models before they can be applied in real practical work in the future. The use of the generated diagnostic models could be limited but it may provide a pattern for CRC screening with combined biomarkers.

## 5 CONCLUSIONS

Our results provide evidence that *F. nucleatum* could be a useful biomarker for CRC. Combining fecal *F. nucleatum* abundance with other clinical markers such as FOB, TRF, CEA, and CA19‐9, as well as personal characteristics such as gender and age have a significantly improved performance for CRC screening in the population. The combination *F. nucleatum* + FOB with gender and age may be an effective method for clinical application. This combined biomarker strategy can help to identify candidates with a high risk of CRC for further diagnostic colonoscopy.

## AUTHOR CONTRIBUTIONS

**Ran Zhao:** Data curation (equal); formal analysis (lead); funding acquisition (equal); methodology (lead); validation (equal); writing—original draft (lead); writing—review and editing (lead). **Dongge Xia:** Data curation (lead); formal analysis (equal); methodology (lead); resources (equal); writing—original draft (equal); writing—review and editing (equal). **Yingwei Chen:** Data curation (equal); formal analysis (equal); methodology (lead); writing—original draft (equal); writing—review and editing (equal). **Zhentian Kai:** Formal analysis (equal); software (lead); writing—original draft (equal); writing—review and editing (equal). **Fangying Ruan:** Data curation (equal); formal analysis (equal); writing—original draft (equal); writing—review and editing (equal). **Chaoran Xia:** Data curation (equal); formal analysis (equal); resources (equal); writing—review and editing (equal). **Jingkai Gong:** Data curation (equal); methodology (equal); writing—original draft (equal). **Jun Wu:** Supervision (equal); writing—review and editing (equal). **Xueliang Wang:** Funding acquisition (lead); methodology (equal); supervision (lead); writing—review and editing (equal).

## FUNDING INFORMATION

This work was supported by the Natural Science Foundation of Shanghai (Grant No: 22ZR1454400), Talents Scheme of Shanghai Center for Clinical Laboratory (Grant No: 2022RCJH‐01), Special General Projects of Clinical Research in Health Industry of Shanghai Municipal Health Commission (Grant No: 202140388), and Youth Medical Talents–Clinical Laboratory Practitioner Program of Shanghai “Rising Stars of Medical Talents” Youth Development Program.

## CONFLICT OF INTEREST STATEMENT

The authors declare that the research was conducted in the absence of any commercial or financial relationships that could be construed as a potential conflict of interest.

## Data Availability

The data that support the findings of this study are available from the corresponding author upon reasonable request.

## References

[1] Dekker E , Tanis PJ , Vleugels JLA , Kasi PM , Wallace MB . Colorectal cancer. Lancet. 2019;394(10207):1467‐1480. 31631858 10.1016/S0140-6736(19)32319-0

[2] Brody H . Colorectal cancer. Nature. 2015;521(7551):S1. 25970450 10.1038/521S1a

[3] Brenner H , Kloor M , Pox CP . Colorectal cancer. Lancet. 2014;383(9927):1490‐1502. 24225001 10.1016/S0140-6736(13)61649-9

[4] Miller KD , Nogueira L , Devasia T , et al. Cancer treatment and survivorship statistics, 2022. CA Cancer J Clin. 2022;72:409‐436. 35736631 10.3322/caac.21731

[5] Hiom SC . Diagnosing cancer earlier: reviewing the evidence for improving cancer survival. Br J Cancer. 2015;112 Suppl 1(Suppl 1):S1‐S5. 25734391 10.1038/bjc.2015.23 PMC4385969

[6] Lakemeyer L , Sander S , Wittau M , Henne‐Bruns D , Kornmann M , Lemke J . Diagnostic and prognostic value of CEA and CA19‐9 in colorectal cancer. Diseases. 2021;9(1):21. 33802962 10.3390/diseases9010021 PMC8006010

[7] Gao Y , Wang J , Zhou Y , Sheng S , Qian SY , Huo X . Evaluation of serum CEA, CA19‐9, CA72‐4, CA125 and ferritin as diagnostic markers and factors of clinical parameters for colorectal cancer. Sci Rep. 2018;8(1):2732. 29426902 10.1038/s41598-018-21048-y PMC5807317

[8] Shaukat A , Levin TR . Current and future colorectal cancer screening strategies. Nat Rev Gastroenterol Hepatol. 2022;19(8):521‐531. 35505243 10.1038/s41575-022-00612-y PMC9063618

[9] Niedermaier T , Balavarca Y , Brenner H . Stage‐specific sensitivity of fecal immunochemical tests for detecting colorectal cancer: systematic review and meta‐analysis. Am J Gastroenterol. 2020;115(1):56‐69. 31850933 10.14309/ajg.0000000000000465 PMC6946106

[10] Duffy MJ . Use of biomarkers in screening for cancer. Adv Exp Med Biol. 2015;867:27‐39. 26530358 10.1007/978-94-017-7215-0_3

[11] Hirata I . Evaluation of the usefulness of the simultaneous assay of fecal hemoglobin (Hb) and transferrin (Tf) in colorectal cancer screening‐for the establishment of the Hb and Tf two‐step cutoff assay (HTTC assay). Diagnosis. 2020;7(2):133‐139. 31472060 10.1515/dx-2019-0049

[12] Sawayama H , Miyamoto Y , Hiyoshi Y , et al. Preoperative transferrin level is a novel prognostic marker for colorectal cancer. Ann Gastroenterol Surg. 2021;5(2):243‐251. 33860145 10.1002/ags3.12411 PMC8034684

[13] Chen JG , Cai J , Wu HL , et al. Colorectal cancer screening: comparison of transferrin and immuno fecal occult blood test. World J Gastroenterol. 2012;18(21):2682‐2688. 22690078 10.3748/wjg.v18.i21.2682 PMC3370006

[14] Krigel A , Wan DW . Colonoscopy after a positive stool‐based test for colon cancer screening: moving toward a better understanding of what to expect. Cancer Prev Res (Phila). 2022;15(7):417‐418. 35788831 10.1158/1940-6207.CAPR-22-0213

[15] Deschoolmeester V , Baay M , Specenier P , Lardon F , Vermorken JB . A review of the most promising biomarkers in colorectal cancer: one step closer to targeted therapy. Oncologist. 2010;15(7):699‐731. 20584808 10.1634/theoncologist.2010-0025 PMC3228001

[16] de Vos WM , Tilg H , Van Hul M , Cani PD . Gut microbiome and health: mechanistic insights. Gut. 2022;71(5):1020‐1032. 35105664 10.1136/gutjnl-2021-326789 PMC8995832

[17] Zhang YJ , Li S , Gan RY , Zhou T , Xu DP , Li HB . Impacts of gut bacteria on human health and diseases. Int J Mol Sci. 2015;16(4):7493‐7519. 25849657 10.3390/ijms16047493 PMC4425030

[18] Shang FM , Liu HL . *Fusobacterium nucleatum* and colorectal cancer: a review. World J Gastrointest Oncol. 2018;10(3):71‐81. 29564037 10.4251/wjgo.v10.i3.71 PMC5852398

[19] Chen Y , Chen Y , Zhang J , et al. *Fusobacterium nucleatum* promotes metastasis in colorectal cancer by activating autophagy signaling via the upregulation of CARD3 expression. Theranostics. 2020;10(1):323‐339. 31903123 10.7150/thno.38870 PMC6929621

[20] Brennan CA , Garrett WS . *Fusobacterium nucleatum*–symbiont, opportunist and oncobacterium. Nat Rev Microbiol. 2019;17(3):156‐166. 30546113 10.1038/s41579-018-0129-6 PMC6589823

[21] Yu T , Guo F , Yu Y , et al. *Fusobacterium nucleatum* promotes chemoresistance to colorectal cancer by modulating autophagy. Cell. 2017;170(3):548‐63.e16. 28753429 10.1016/j.cell.2017.07.008 PMC5767127

[22] Ranjbar M , Salehi R , Haghjooy Javanmard S , et al. The dysbiosis signature of *Fusobacterium nucleatum* in colorectal cancer‐cause or consequences? A systematic review. Cancer Cell Int. 2021;21(1):194. 33823861 10.1186/s12935-021-01886-z PMC8025348

[23] Kostic AD , Chun E , Robertson L , et al. *Fusobacterium nucleatum* potentiates intestinal tumorigenesis and modulates the tumor‐immune microenvironment. Cell Host Microbe. 2013;14(2):207‐215. 23954159 10.1016/j.chom.2013.07.007 PMC3772512

[24] Zhang X , Zhu X , Cao Y , Fang JY , Hong J , Chen H . Fecal *Fusobacterium nucleatum* for the diagnosis of colorectal tumor: a systematic review and meta‐analysis. Cancer Med. 2019;8(2):480‐491. 30636375 10.1002/cam4.1850 PMC6382715

[25] Komor MA , Bosch LJ , Coupé VM , et al. Proteins in stool as biomarkers for non‐invasive detection of colorectal adenomas with high risk of progression. J Pathol. 2020;250(3):288‐298. 31784980 10.1002/path.5369 PMC7065084

[26] Nikolaou S , Qiu S , Fiorentino F , Rasheed S , Tekkis P , Kontovounisios C . Systematic review of blood diagnostic markers in colorectal cancer. Tech Coloproctol. 2018;22(7):481‐498. 30022330 10.1007/s10151-018-1820-3 PMC6097737

[27] Tepus M , Yau TO . Non‐invasive colorectal cancer screening: an overview. Gastrointest Tumors. 2020;7(3):62‐73. 32903904 10.1159/000507701 PMC7445682

[28] Brenner H , Stock C , Hoffmeister M . Effect of screening sigmoidoscopy and screening colonoscopy on colorectal cancer incidence and mortality: systematic review and meta‐analysis of randomised controlled trials and observational studies. BMJ. 2014;348:g2467. 24922745 10.1136/bmj.g2467 PMC3980789

[29] Rees CJ , Bevan R , Zimmermann‐Fraedrich K , et al. Expert opinions and scientific evidence for colonoscopy key performance indicators. Gut. 2016;65(12):2045‐2060. 27802153 10.1136/gutjnl-2016-312043 PMC5136701

[30] Vukobrat‐Bijedic Z , Husic‐Selimovic A , Sofic A , et al. Cancer antigens (CEA and CA 19‐9) as markers of advanced stage of colorectal carcinoma. Med Arch. 2013;67(6):397‐401. 25568506 10.5455/medarh.2013.67.397-401 PMC4272469

[31] Lech G , Słotwiński R , Słodkowski M , Krasnodębski IW . Colorectal cancer tumour markers and biomarkers: recent therapeutic advances. World J Gastroenterol. 2016;22(5):1745‐1755. 26855534 10.3748/wjg.v22.i5.1745 PMC4724606

[32] Dickinson BT , Kisiel J , Ahlquist DA , Grady WM . Molecular markers for colorectal cancer screening. Gut. 2015;64(9):1485‐1494. 25994221 10.1136/gutjnl-2014-308075 PMC4765995

[33] Liang Q , Chiu J , Chen Y , et al. Fecal bacteria act as novel biomarkers for noninvasive diagnosis of colorectal cancer. Clin Cancer Res. 2017;23(8):2061‐2070. 27697996 10.1158/1078-0432.CCR-16-1599

[34] Tunsjø HS , Gundersen G , Rangnes F , Noone JC , Endres A , Bemanian V . Detection of *Fusobacterium nucleatum* in stool and colonic tissues from Norwegian colorectal cancer patients. Eur J Clin Microbiol Infect Dis. 2019;38(7):1367‐1376. 31025134 10.1007/s10096-019-03562-7

[35] Mannucci A , Zuppardo RA , Rosati R , Leo MD , Perea J , Cavestro GM . Colorectal cancer screening from 45 years of age: thesis, antithesis and synthesis. World J Gastroenterol. 2019;25(21):2565‐2580. 31210710 10.3748/wjg.v25.i21.2565 PMC6558439

